# Protective and Therapeutic Effects of Orlistat on Metabolic Syndrome and Oxidative Stress in High-Fat Diet-Induced Metabolic Dysfunction-Associated Fatty Liver Disease (MAFLD) in Rats: Role on Nrf2 Activation

**DOI:** 10.3390/vetsci8110274

**Published:** 2021-11-12

**Authors:** Zaida Zakaria, Zaidatul Akmal Othman, Joseph Bagi Suleiman, Nur Asyilla Che Jalil, Wan Syaheedah Wan Ghazali, Mahaneem Mohamed

**Affiliations:** 1 Department of Physiology, School of Medical Sciences, Universiti Sains Malaysia, Kubang Kerian 16150, Kelantan, Malaysia; zaida_zakaria@student.usm.my (Z.Z.); zaidaakmal@unisza.edu.my (Z.A.O.); syaheeda@usm.my (W.S.W.G.); 2 Unit of Physiology, Universiti Sultan Zainal Abidin, Kuala Terengganu 20400, Terengganu, Malaysia; 3 Department of Science Laboratory Technology, Akanu Ibiam Federal Polytechnic, Unwana P.M.B 1007, Ebonyi State, Nigeria; bagisuleiman@student.usm.my; 4 Department of Pathology, School of Medical Sciences, Universiti Sains Malaysia, Kubang Kerian 16150, Kelantan, Malaysia; asyilla@usm.my; 5 Unit of Integrative Medicine, School of Medical Sciences, Universiti Sains Malaysia, Kubang Kerian 16150, Kelantan, Malaysia

**Keywords:** Keap1, metabolic syndrome, NAFLD, Nrf2, orlistat, oxidative stress parameters

## Abstract

Metabolic dysfunction-associated fatty liver disease (MAFLD) is an excessive buildup of liver lipids closely associated with various kinds of undesirable metabolic effects and oxidative stress. We aimed to investigate the protective and therapeutic effects of orlistat on metabolic syndrome and oxidative stress parameters in high-fat diet (HFD) induced-MAFLD rats. Twenty-four male *Sprague-Dawley* rats were randomly divided into four groups (*n* = 6/group), i.e., Normal control (N), HFD, HFD + orlistat (HFD + O) (10 mg/kg/day administered concomitantly for 12 weeks as a protective model), and obese+orlistat (OB + O) (10 mg/kg/day administered 6 weeks after induction of obesity as a therapeutic model) groups. After 12 weeks, the HFD group had significantly increased Lee obesity index, serum alanine aminotransferase, aspartate aminotransferase, alkaline phosphatase, total cholesterol, triglyceride, low-density lipoprotein levels, liver total cholesterol and triglyceride levels, insulin resistance and non-alcoholic steatohepatitis (NASH) together with decreased serum high-density lipoprotein level. Additionally, the HFD group also showed increased Nrf2 translocation to the nucleus with high Keap1 expression and increased liver oxidative stress parameters. Orlistat significantly improved all these alterations in HFD rats. We demonstrated that orlistat might have protective and therapeutic effects against HFD-induced MAFLD rats by its activation on Nrf2 signaling pathway, which subsequently improved metabolic syndrome and oxidative stress parameters.

## 1. Introduction

Non-alcoholic fatty liver disease (NAFLD) is linked with obesity and imbalance dietary intake such as excessive intake of high-fat diet (HFD). It is a common liver disorder in developed countries, affecting 75–100 million Americans in 2017 [1]. NAFLD is recognized as an abnormal accumulation of triglycerides (TGs) in the hepatocytes (surpassing 5 to 10% of the liver weight) and comprises a spectrum of histological findings that range from simple steatosis (presence of macro-vesicular steatosis only) to non-alcoholic steatohepatitis (NASH) (presence of macro-vesicular steatosis with hepatocyte ballooning and with/without lobular inflammation) [2], advanced fibrosis, cirrhosis, hepatocellular carcinoma, and liver-related mortality [3,4].

Many studies have been reported the relation between NAFLD and the main features of metabolic syndrome [5,6], which include obesity [7,8], hyperglycemia [9], hyperlipidemia [10,11], hypertension [12,13], insulin resistance [14,15], and a proinflammatory state [16]. Hence, this condition has been newly re-named as metabolic dysfunction-associated fatty liver disease (MAFLD) [17,18] and the new acronym MAFLD is used to replace the older term NAFLD throughout this study. MAFLD has been recognized as the hepatic manifestation of metabolic syndrome [19] which is linked to increased production of reactive oxygen species (ROS), accompanied with augmented oxidative stress and attenuation of antioxidant enzymes activities in the liver tissue [20]. Furthermore, the latest studies have come out with evidence which support the idea that increased oxidative stress could also be one of the key players in metabolic syndrome, which manifests many life-threating diseases including atherosclerosis [21], hypertension [22], and type 2 diabetes [23].

Nuclear factor erythroid 2-related factor 2 (Nrf2) is a key regulator of antioxidant signaling, abundantly expressed in many tissues and cell types, and it is the most important signaling pathway in the defense against oxidative stress in the cell. Nrf2 is predominantly identified in the cytoplasm through its association with Kelch-like ECH-associated protein 1 (Keap1) and the actin cytoskeleton [24]. Under quiescent condition, the actions of Keap1 on Nrf2 is for polyubiquitination and degradation, which subsequently exerts inhibition towards Nrf2. Upon exposure to high oxidative stress condition, Keap1 becomes oxidized while Nrf2 is then dissociated from Keap1 and translocated into the nucleus, where it eventually activates the expression of the antioxidant response element (ARE), which is found in the promoter region of genes encoded for antioxidant enzymes and stimulates the transcriptions of various anti-oxidative enzyme genes such as superoxide dismutase (SOD), glutathione peroxidase (GPx), catalase (CAT), glutathione reductase (GR), glutamate cysteine ligase (GCL), and NAD(P)H/quinone oxidoreductase 1 (NQO1) [25]. Apart from its main function to regulate genes encoded for antioxidant enzymes that are involved in scavenging ROS, Nrf2 also plays a protective role in MAFLD by exerting negative regulation on genes that are involved in lipid accumulation. Previous studies showed that the intake of HFD in Nrf2-null mice resulted in severe hepatic steatosis and substantial inflammation compared to intake of HFD in wild-type mice, which exhibited only mild hepatic steatosis without the presence of inflammation [26,27]. It has been reported that the expression of Nrf2 is reduced in liver tissue of MAFLD animal model [6,28].

Nowadays, the diet composition improvement represents a key factor to enhance the health status and welfare of animals; indeed, within the scientific community, the diet supplementation has been widely accepted as a useful strategy to modulate and/or optimize the biochemical and molecular pathways which orchestrate the metabolic responses of the animal to both physiological and pathological conditions [29,30]. Nevertheless, pharmacological therapy is still warranted especially in certain resistant cases of MAFLD. Orlistat, a potent gastric and pancreatic lipase inhibitor with a weight-reducing effect has been recommended in the treatment of obesity, which is an independent risk factor of MAFLD. Usage of orlistat has succeeded in reducing the absorption of fat in the gastrointestinal tract and hence in blocking the dietary TGs from entering the liver [31]. Several studies have demonstrated that treatment with orlistat ameliorated metabolic variables [31,32,33] and oxidative stress [34,35] in obese rats. Our previous research reported an up-regulation of Nrf2 gene expression in the testis of HFD rats after orlistat intake for 12 weeks [36]. Nevertheless, no finding has been reported on the activation of Nrf2 in the liver after orlistat administration.

Therefore, the beneficial effects of orlistat in ameliorating metabolic syndrome and oxidative stress in MAFLD are still unclear. Herein, we sought to evaluate whether orlistat could improve metabolic syndrome and oxidative stress in HFD-induced MAFLD rats and activate Nrf2/Keap1 signaling pathway to exert its protective and therapeutic effects.

## 2. Materials and Methods

### 2.1. Animal and Treatment

Twenty-four male *Sprague-Dawley* rats aged 8–10 weeks (200–230 g) were procured from Laboratory Animal Research Unit of Universiti Sains Malaysia (USM), Health Campus, Kelantan, Malaysia and acclimatized to the experimental facility for at least a week prior to the experiment. The rats were housed in an animal room at 23 ± 2 °C with relative humidity within 55% to 65% and 12-h light/12-h dark conditions and allowed free access to food and water. After acclimatization, all animals were supplied with either normal pellet food (Altromin Spezialfutter GmbH & Co. KG, Lage, Germany) or HFD, and water ad libitum, and randomly divided into four groups (*n* = 6/group), namely:

1. Normal control (N) group: Received normal pellet food and distilled water for 12 weeks;
2. High-fat diet (HFD) group: Received HFD for 12 weeks;
3. HFD + orlistat (HFD + O) group: Received HFD and orlistat (10 mg/kg of body weight/day) concomitantly for 12 weeks as a protective model;
4. Obese + orlistat (OB + O) group: Received HFD for 6 weeks to induce obesity and received both HFD and orlistat (10 mg/kg of body weight/day) during the last 6 weeks as a therapeutic model.

In the OB + O group, the Lee obesity index was calculated after 6 weeks of HFD administration using a previously reported formula [37]: $\frac{\sqrt[{3}]{body}weight\left(g\right)}{naso-anal\;length\;\left(cm\right)}\times 1000$. The rats with Lee obesity index of more than 315 were considered as obese [38] and were used in the subsequent 6 weeks of the treatment phase with orlistat (Xepa-Soul Pattinson Sdn. Bhd. Melaka, Malaysia). The dose of orlistat (10 mg/kg/day) was applied as reported in a previous study [39]. Meanwhile, HFD consisted of 32 g ghee (Unilever Holdings Sdn. Bhd. Kuala Lumpur, Malaysia), 68 g of crushed normal pellet food, 300 mg of calcium, and 100 IU of vitamin D (Eurobio Sdn. Bhd. Victoria, Australia) as well as 12% of cholesterol powder (Nacalai Tesque, Kyoto, Japan) [34]. HFD was freshly prepared by mixing all the ingredients thoroughly and shaped into smaller pieces and stored overnight at 4 °C before being fed to the animals. The composition of the diets is shown in Table 1 as described by Othman et al. [40].

Body weight and food intake were measured every week and daily, respectively. At the end of the experiment, the Lee obesity index was recalculated. All the animals were starved overnight (12 h) and anaesthetized by ketamine and xylazine (90 mg/kg and 5 mg/kg, IP, respectively). Laparotomy was performed and a blood sample was collected from the posterior vena cava. Blood samples were left at room temperature to allow for coagulation before being centrifuged at 4000 rpm for 15 min at 4 °C to separate the serum (Avanti J-HC, Beckman Coulter, IN, USA). The obtained serum was then transferred into eppendorf tubes and kept at −80 °C until analysis. The liver and adipose tissues (epididymal, peritoneal and perirenal) were harvested and dissected out, then rinsed with normal saline before being weighed using an analytical balance (Denver Instrument Company, Arvada, CO, USA). The liver tissues were weighed and homogenized in 10 volumes of cold phosphate buffer saline (pH 7.4) before being centrifuged at 4000 rpm for 15 min at 4 °C. The supernatant was collected and kept at −80 °C until analysis. The liver tissues were also fixed in 10% formalin for histopathological and immunohistochemical analyses. The experimental protocol was approved by USM Institutional Animal Care and Use Committee (USM IACUC) (No. of Animal Ethics Approval: USM/IACUC/2018(113)(933) and USM/IACUC/2020/(126)(1109)).

### 2.2. Oral Glucose Tolerance Test (OGTT)

OGTT was determined for all animal groups at the end of the 12th week of the experimental period and all the rats were fasted overnight (12 h) prior to the test. Basal blood glucose concentration was measured from the tail vein at time 0 using glucometer (URight TD-4279 Blood Glucose Monitoring System, Taipei, Taiwan). The rats were then administered with glucose (2 g/kg) via oral gavage. Blood samples were collected at 30, 60, 90, and 120 min, following glucose administration.

### 2.3. Measurements of Serum Glucose, Insulin and HOMA-IR

Serum glucose and insulin were determined using rat enzyme-linked immunosorbent assay (ELISA) kits purchased from Qayee-Bio Life Science Co., Ltd., Shanghai, China (Catalog No: QY-E11702) and Elabscience Biotechnology Inc. Co., Ltd. Wuhan, Hubei, China (Catalog No: E-EL-R2466), respectively, according to the manufacturer’s instructions. The homeostasis model assessment for insulin resistance (HOMA-IR) was determined as follows: HOMA-IR = fasting insulin (ng/mL) × fasting blood glucose (mg/dL)/405 [41].

### 2.4. Determination of Lipid and Liver Function Profiles

Serum total cholesterol (TC) was determined via enzymatic-colorimetric method using assay kit (Architect c total cholesterol kit, Abbott, IL, USA) according to the manufacturer’s instruction where quinoneimine was formed as an end product of hydrogen peroxide (sensitivity 18.26 mmol/L and coefficient of variation, CV ≤ 3%). Meanwhile, serum TG was evaluated using an assay kit (Architect c triglyceride kit, Abbott, IL, USA) according to the manufacturer’s instruction, which produced fatty acids and glycerol from hydrolysis of lipase (CV ≤ 5% and sensitivity 16.05). The levels of both TC and TG were determined with absorbance at 500 nm.

Serum low-density lipoprotein (LDL) was measured using the formula as described by a previous study [42]: LDL (mmol/L) = (TC – HDL − TG)/5. Similarly, high-density lipoprotein (HDL) was determined via removal of LDL and chylomicrons, as well as very-low density lipoprotein by cholesterol esterase, catalase, and cholesterol oxidase using commercial kit (Biosino Bio-Technology and Science Inc., Beijing, China) according to manufacturer’s instruction (sensitivity up to 2.6 mmol/L) with absorbance at 600 nm.

Whereas, aspartate aminotransferase (AST), alanine aminotransferase (ALT) and alkaline phosphatase (ALP) were evaluated using the International Federation of Clinical Chemistry (IFCC) method, which measured the catalytic concentration of reagent enzymes and the contaminants. All liver enzymes were determined using Abbott-Achitect Ci8200, Abbott Park, IL, USA.

### 2.5. Liver Lipid Content

The levels of liver TC and TG were measured via rat ELISA kits purchased from Qayee-Bio Life Science Co., Ltd. (Shanghai, China), catalog no: QY-E10860 and QY-E11395, respectively according to the manufacturer’s instructions.

### 2.6. Analysis of Liver Oxidative Stress Markers

Lipid peroxidation was assessed in the liver as thiobarbituric acid reactive substances (TBARS) using a previously reported method [43] The reaction changes were measured with absorbance at 532 nm with tetraethoxypropane as the standard. The level of nitric oxide (NO) was evaluated with a commercially available kit from Elabscience Biotechnology Inc. Co., Ltd. (Wuhan, China) (Catalog no: E-BC-K035-M) according to the manufacturer’s instruction. Protein oxidation in the rats was determined based on the concentration of protein carbonyl (PCO) as described by a previous study [44]. The reaction between protein carbonyls with 2,4-dinitrophenylhydrazine (DNPH) resulted in the production of colored complex hydrazone. Furthermore, the level of hydrazone was measured spectrophotometrically at 370 nm. Results were normalized with protein levels using commercially available protein assay kit obtained from Thermo Scientific (Rockford, IL, USA).

### 2.7. Analysis of Liver Antioxidant Enzyme Activities

Liver SOD activity was evaluated based on the percentage reduction of nitro tetrazolium blue using a previously described method [45] which was quantified spectrophotometrically at 560 nm. In addition, the activity of CAT was assessed according to the previously described method [46], which was based on hydrogen peroxide decomposition with molybdate and forms a yellowish complex. Estimation of GPx activity was performed using a method based on glutathione oxidation by hydrogen peroxide as described by a previous study [47]. The activity of glutathione S-transferase (GST) was assayed based on glutathione (GSH) conjugation to 1-chloro-2,4-dinitrobenzene as the substrate [48]. GR activity in the liver was determined based on glutathione disulphide reduction in the presence of NADPH to GSH and NADP+, which was catalyzed by GR as described by a previous study [49]. All the results were normalized with protein levels.

### 2.8. Analysis of Liver Glutathione and Total Antioxidant Capacity

Determination of GSH concentration was done based on the reaction between 5,5′-dithiobis-2-nitrobenzoic acid with the sulfhydryl group of GSH to produce 5-thio-2-nitobenzoic acid as described by a previous study [50]. The liver total antioxidant capacity (TAC) was measured in this study by estimating the combined antioxidant activities of all its constituents in both lipid soluble and aqueous forms. Antioxidants suppressed the formation of TBARS, and this reaction was measured spectrophotometrically at 532 nm [51]. All the results were normalized with protein levels.

### 2.9. Immunohistochemical Detections of Nrf2 and Keap1 Expressions

Immunohistochemistry (IHC) for Nrf2 and Keap1 were performed after deparaffinization, rehydration, and followed by antigen retrieval with tris-EDTA buffer containing 0.05% Tween 20 using the pressure cooker method. Next, the liver sections were incubated in 3% hydrogen peroxide (diluted in phosphate buffer saline) to block endogenous peroxidase activity and then washed with distilled water followed by Tris-buffered saline containing 0.05% Tween 20 (TBST). Sections were then incubated in the following rabbit polyclonal primary antibodies: Nrf2 (Cloud-Clone Corp, Katy, TX, USA) (1:100) and Keap1 (Cloud-Clone Corp, Katy, TX, USA) (1:100) antibodies at 4 °C overnight and washed with TBST. Then, sections were incubated with Dako EnVision System Labelled Polymer-HRP (Agilent Technologies, Inc., Santa Clara, CA, USA) containing goat anti-rabbit secondary antibody at room temperature for 1 h and washed with TBST. Sections were incubated with Dako DAB + substrate chromogen (Agilent Technologies, Inc., Santa Clara, CA, USA) (1:1) mixed solution for 5 min at room temperature before being washed under running water for 5 min. Sections were counterstained with Harris hematoxylin (Merck, Darmstadt, Germany). The percentage of positive staining in the cytoplasm and nucleus were evaluated.

IHC staining was assessed by two independent pathologists who were blinded to the experimental data. In the present study, the scoring patterns for Nrf2 and Keap1 were evaluated as described by a previous study [52] as follows: score 0, negative staining for all cells; score 1+, weakly positive staining in <10% of cells; score 2+, moderate to strong positive covering between 10 to 50% of cells; score 3+, strongly positive staining including >50% cells. Light microscope (Olympus BX41, Tokyo, Japan) with a digital camera (Olympus XC50, Tokyo, Japan) were used to capture all the images at 40× magnification.

### 2.10. Histopathological Analysis

The formalin-fixed liver tissues were embedded and sectioned at 3 µm thicknesses. The sections were then stained with Harris hematoxylin (Merck, Darmstadt, Germany) and eosin (Merck, Darmstadt, Germany) (H&E) for visualization of hepatic tissue architecture, morphological changes, and inflammatory cell infiltration. All histopathological examinations were graded according to the NALFD activity score (NAS) grading system for rodent [53,54] by two independent pathologists, who were blinded to the experimental and serological data. A NAS score of ≥5 is diagnosed as NASH and a score of ≤3 is referred to as non-NASH. The stained sections were photographed using a light microscope (Olympus BX41, Tokyo, Japan) with a digital camera (Olympus XC50, Tokyo, Japan) at 40× magnification.

### 2.11. Statistical Analysis

Statistical analysis was evaluated using GraphPad Prism version 8 (GraphPad Software Inc., San Diego, CA, USA). The normality and variance of the data were checked using the Shapiro-Wilk and D’Agostino-Pearson Omnibus normality test, respectively. The differences among the groups were analyzed by the one-way ANOVA test followed by Tukey’s post-hoc test. All values are presented as means ± standard error of the means (SEM) with *p* < 0.05 as the criterion used for statistical significance.

## 3. Results

### 3.1. Lee Obesity Index, Body Weight Gain, Food Intake, and Calorie Intake

Lee obesity index and body weight gain are essential parameters for determining the effects of HFD on the classification and development of obesity, respectively. Rats in the HFD group demonstrated significant increased Lee obesity index as well as body weight gain compared to rats in the N group. Orlistat administrations in both protective and therapeutic groups significantly reduced the Lee obesity index as well as body weight gain in comparison with the HFD group. The average food intake among the groups were not significantly changed. However, all groups which received the HFD had significantly higher calories than the N group, but no significant changes were observed between the HFD fed groups (Table 2).

### 3.2. Effects of Orlistat on Liver and Adipose Tissue Weights

Absolute liver weight, relative liver weight as well as adipose tissue weight from rats in each group are shown in Table 3. HFD group showed significantly higher absolute liver weight, relative liver weight, epididymal, peritoneal, perirenal, and total adipose tissue weights in comparison to the N group. Orlistat administration in both HFD + O and OB + O groups significantly decreased all these parameters in comparison with the HFD group.

### 3.3. Effects of Orlistat on Oral Glucose Tolerance Test (OGTT)

**Figure 1:**
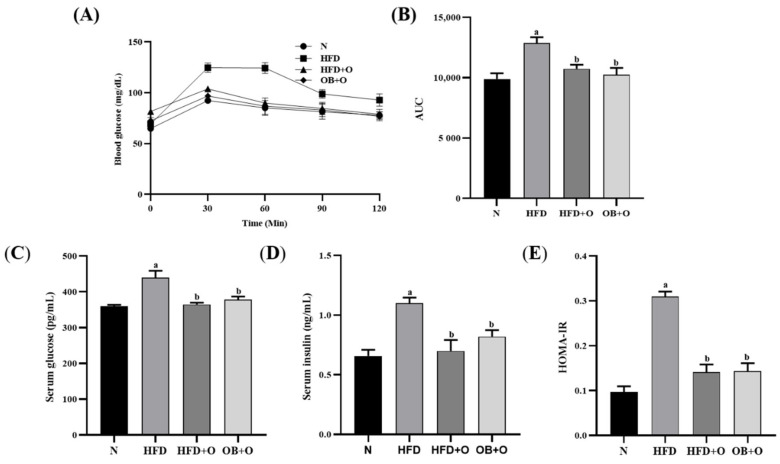
Insulin sensitivity in HFD-induced MAFLD rats. N, normal control; HFD, high-fat diet; HFD + O, high-fat diet + orlistat 10 mg/kg/day (protective model); OB + O, obese + orlistat 10 mg/kg/day (therapeutic model). (**A**) Oral glucose tolerance test (OGTT); (**B**) Area under the curve (AUC); (**C**) Serum glucose; (**D**) Serum insulin; (**E**) HOMA-IR. Data are expressed as mean ± SEM, *n* = 6/group. One-way ANOVA, followed by Tukey post-hoc test. ^a^ *p* < 0.05 vs. N group, ^b^ *p* < 0.05 vs. HFD group.

As shown in Figure 1A, after 12-weeks of the experimental period, the basal blood glucose levels were similar among the groups and increased at 30 min following glucose intake. Nevertheless, the blood glucose levels were normalized within 60–120 min in all groups except for rats in the HFD group. After 120 min of glucose intake, the HFD group showed an increment in the blood glucose level, whereas orlistat administration in both protective and therapeutic groups showed reduction in the blood glucose level. The analysis of area under curve (AUC) in Figure 1B shows significant increased AUC value in the HFD group in comparison with the N group. Orlistat administration in the HFD + O and OB + O groups significantly normalized the AUC value.

### 3.4. Effects of Orlistat on Insulin Sensitivity

The levels of serum glucose and insulin in HFD group were significantly elevated than in the N group (Figure 1C,D). Furthermore, the HFD group demonstrated a higher HOMA-IR index due to the continuous and prolonged intake of HFD, which resulted in a substantial reduction in insulin sensitivity. In contrast, the levels of glucose and insulin as well as HOMA-IR index were significantly reduced following orlistat administrations in both protective and therapeutic models (Figure 1E).

### 3.5. Effects of Orlistat on Lipid Profiles

**Figure 2:**
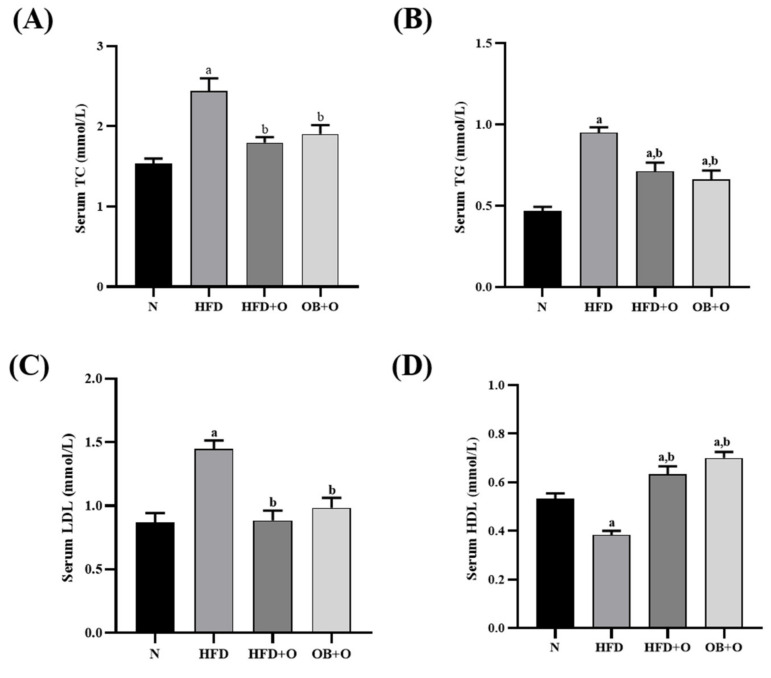
Serum lipid profiles in HFD-induced MAFLD rats. N, normal control; HFD, high-fat diet; HFD + O, high-fat diet+orlistat 10 mg/kg/day (protective model); OB + O, obese+orlistat 10 mg/kg/day (therapeutic model); TC, total cholesterol; TG, triglyceride; LDL, low-density lipoprotein; HDL, high-density lipoprotein. (**A**) Serum TC; (**B**) Serum TG; (**C**) Serum LDL; (**D**) Serum HDL. Data are expressed as mean ± SEM, *n* = 6/group. One-way ANOVA, followed by Tukey post-hoc test. ^a^ *p* < 0.05 vs. N group, ^b^ *p* < 0.05 vs. HFD group.

Serum levels of TC, TG, and LDL were significantly augmented whereas the level of HDL was significantly reduced in the HFD group, relative to the N group (Figure 2A–D). However, orlistat administration in both HFD + O and OB + O groups significantly reduced the levels of TC, TG, and LDL, relative to the HFD group. Moreover, the intake of orlistat in both groups also increased the level of HDL in comparison with HFD and N groups.

### 3.6. Effects of Orlistat on Liver Lipid Content

**Figure 3:**
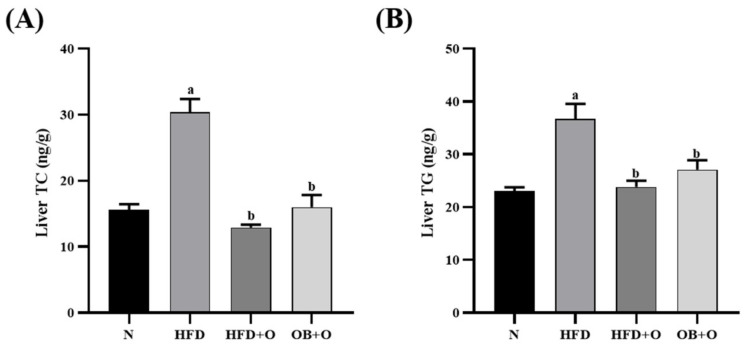
Liver lipid content in HFD-induced MAFLD rats. N, normal control; HFD, high-fat diet; HFD + O, high-fat diet+orlistat 10 mg/kg/day (protective model); OB + O, obese + orlistat 10 mg/kg/day (therapeutic model); TC, total cholesterol; TG, triglyceride. (**A**) TC; (**B**) TG. Data are expressed as mean ± SEM, *n* = 6/group. One-way ANOVA, followed by Tukey post-hoc test. ^a^ *p* < 0.05 vs. N group, ^b^ *p* < 0.05 vs. HFD group.

Liver lipid contents were evaluated in this study as shown in Figure 3. Levels of liver TC and TG were significantly elevated in the HFD group when compared to those in the N group. However, these parameters were significantly reduced following orlistat administration in both protective and therapeutic groups.

### 3.7. Effects of Orlistat on Liver Functions

**Figure 4:**
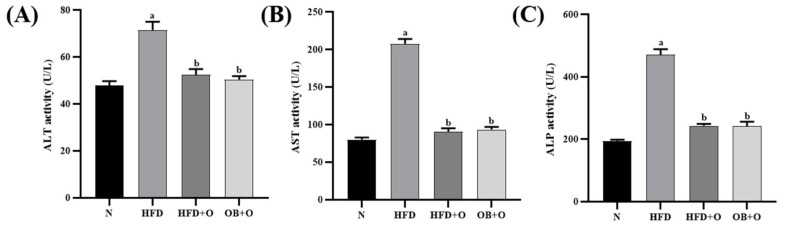
Liver functions in HFD-induced MAFLD rats. N, normal control; HFD, high-fat diet; HFD + O, high-fat diet+orlistat 10 mg/kg/day (protective model); OB + O, obese + orlistat 10 mg/kg/day (therapeutic model); ALT, alanine aminotransferase; AST, aspartate aminotransferase; ALP, alkaline phosphatase. (**A**) ALT; (**B**) AST; (**C**) ALP. Data are expressed as mean ± SEM, *n* = 6/group. One-way ANOVA, followed by Tukey post-hoc test. ^a^ *p* < 0.05 vs. N group, ^b^ *p* < 0.05 vs. HFD group.

Liver injury was evaluated by assessing the activities of enzyme markers of liver function. Changes in liver enzymes are demonstrated in Figure 4. The results showed that the intake of HFD significantly increased serum ALT, AST, and ALP activities in the HFD group, in comparison with the N group. In contrast, the intake of orlistat normalized these liver enzymes activities as demonstrated in both HFD + O and OB + O groups compared to the animals of HFD group.

### 3.8. Effects of Orlistat on Liver Oxidative Stress Markers

Oxidative stress condition was assessed by determining the levels of TBARS, NO, and PCO in the liver. In the present study, HFD feeding resulted in significant increases in TBARS, NO, and PCO levels in the HFD group when compared to the N group. On the contrary, significant decreases in TBARS, NO, and PCO concentrations were observed in both orlistat groups (HFD + O and OB + O), in comparison with the HFD group (Table 4).

### 3.9. Effects of Orlistat on Liver Antioxidant Enzyme Activities

SOD, CAT, GPx, GST, and GR are naturally produced cellular antioxidants which are accountable for decreasing the oxidative stress. The activities of SOD, CAT, GPx, GST, and GR were significantly reduced in HFD group, in comparison with the N group. The intake of orlistat in both protective and therapeutic groups significantly restored all these antioxidant enzymes activities compared to the HFD group (Table 4).

### 3.10. Effects of Orlistat on Glutathione and Total Antioxidant Capacity

In this study, GSH and TAC were significantly lowered in HFD group, in comparison with the N group. However, GSH and TAC were significantly augmented in both protective and therapeutic groups compared to HFD group.

### 3.11. Effects of Orlistat on Immunohistochemical Analysis of Nrf2 and Keap1 Expressions

**Figure 5:**
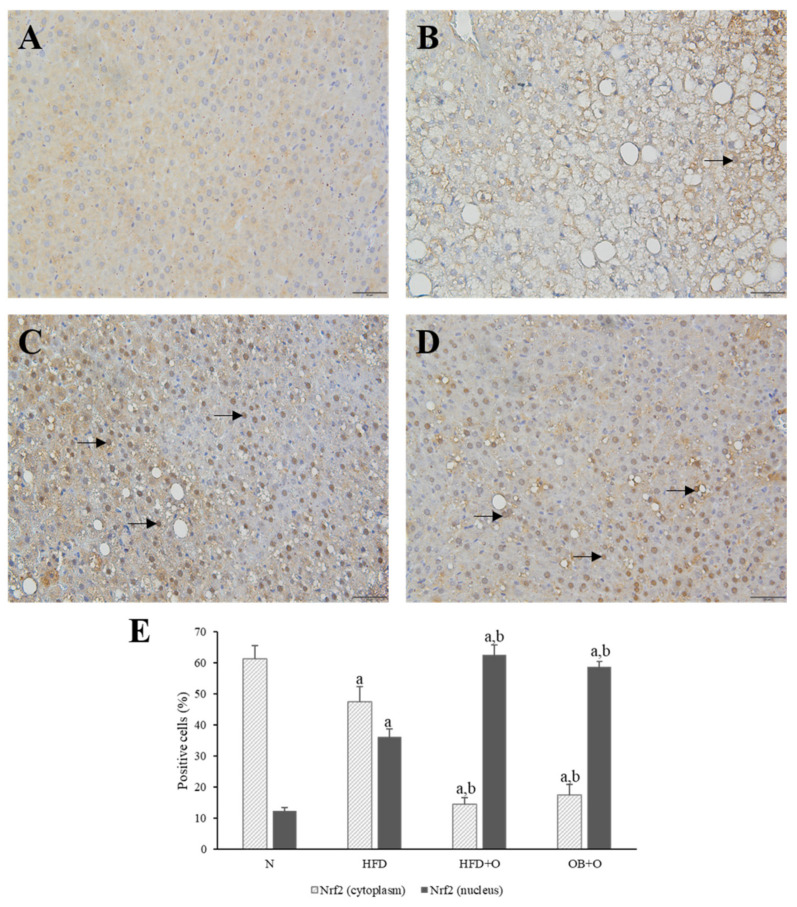
(**A**–**E**). Immunohistochemical staining of Nrf2 expression in liver sections. N, normal control; HFD, high-fat diet; HFD + O, high-fat diet + orlistat 10 mg/kg/day (protective model); OB + O, obese + orlistat 10 mg/kg/day (therapeutic model); Nrf2, nuclear factor erythroid 2-related factor 2. (**A**) N group shows accumulation of Nrf2 in the cytoplasm than in the nuclei. (**B**) More Nrf2 was concentrated in the nuclei of the HFD group than in the N group. (**C**,**D**) This concentration morphology was more apparent after orlistat administration in the HFD + O and OB + O groups. Magnification, ×400. Arrows indicate nucleus-positive cells. (**E**) Quantification of Nrf2-positive cells (%). Data are expressed as mean ± SEM, *n* = 6/group. One-way ANOVA, followed by Tukey post-hoc test. ^a^ *p* < 0.05 vs. N group, ^b^ *p* < 0.05 vs. HFD group.

**Figure 6:**
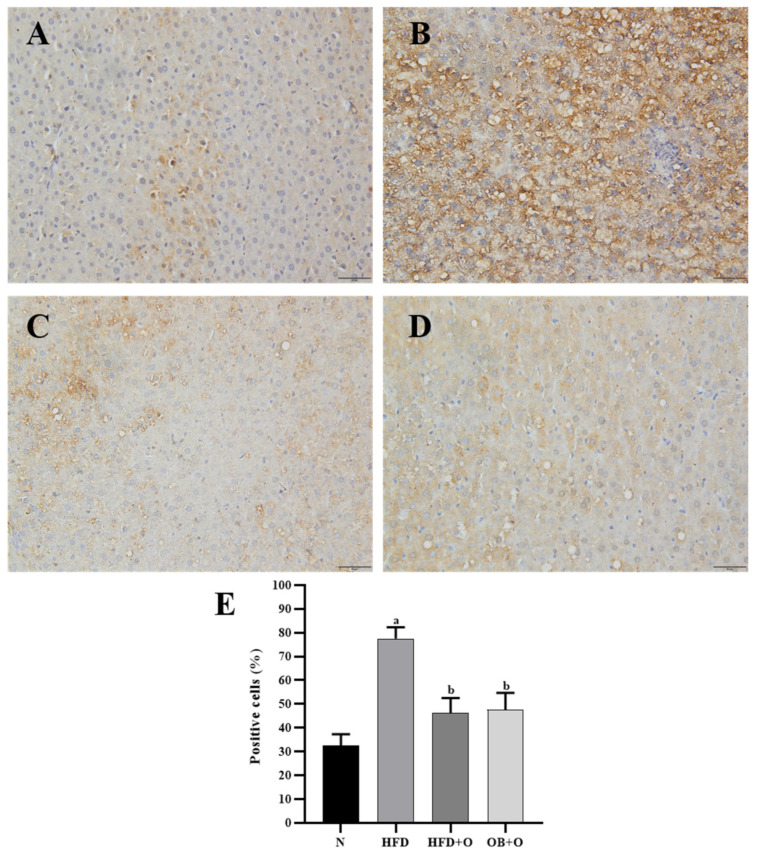
(**A**–**E**). Immunohistochemical staining of Keap1 expression in liver sections. N, normal control; HFD, high-fat diet; HFD + O, high-fat diet + orlistat 10 mg/kg/day (protective model); OB + O, obese+orlistat 10 mg/kg/day (therapeutic model); Keap1, Kelch-like ECH-associated protein 1. (**A**) The N group shows less accumulation of Keap1 in the cytoplasm. (**B**) More Keap1 was concentrated in the cytoplasm of the HFD group than in the N group. (**C**,**D**) This concentration morphology was reduced after orlistat administration in the HFD + O and OB + O groups. Magnification, ×400. (**E**) Quantification of Keap1-positive cells (%). Data are expressed as mean ± SEM, *n* = 6/group. One-way ANOVA, followed by Tukey post-hoc test. ^a^ *p* < 0.05 vs. N group, ^b^ *p* < 0.05 vs. HFD group.

Immunohistochemistry localization was performed to assess the expressions of Nrf2 and Keap1 in the liver tissue sections. As shown in Figure 5, Nrf2 protein was predominantly located in the cytoplasm. Results revealed low expression of Nrf2 in the cytoplasm and high expression of Nrf2 in the nuclei of the HFD group in comparison with the N group. The administration of orlistat led to low Nrf2 expression in the cytoplasm, whereas high Nrf2 expression in the nuclei was observed in HFD + O and OB + O groups when compared to the HFD group, which indicate a higher translocation of cytoplasmic Nrf2 into the nucleus (Figure 5A–E). In addition, Keap1 expression, which is mainly present in the cytoplasm, was significantly augmented in the HFD group in comparison with the N group, and the intake of orlistat in both groups reduced the Keap1 expression in comparison with HFD group, indicating the loss of Keap1 inhibitory effect on Nrf2 in these groups (Figure 6A–E).

### 3.12. Effects of Orlistat on MAFLD and Analysis of NAS

**Figure 7:**
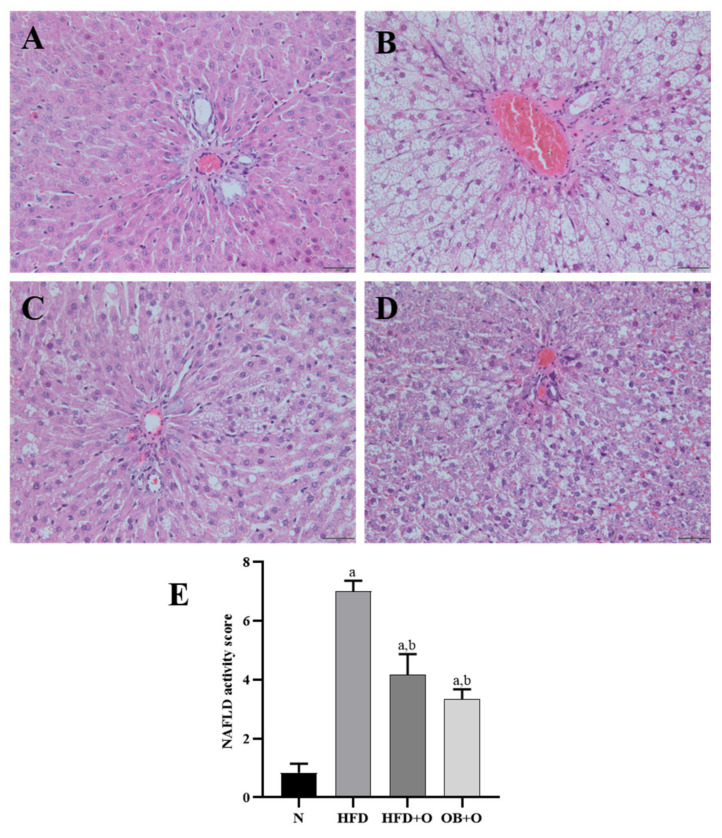
(**A**–**E**). Photomicrographs of the liver using hematoxylin and eosin staining. N, normal control; HFD, high-fat diet; HFD + O, high-fat diet + orlistat 10 mg/kg/day (protective model); OB + O, obese+orlistat 10 mg/kg/day (therapeutic model). (**A**) The N group shows normal liver architecture. (**B**) The HFD group shows severe hepatic steatosis and marked inflammatory cells around the portal triad. (**C**,**D**) The HFD + O and OB + O groups show reduction of fat deposition and inflammation. (H&E, magnification, ×400). (**E**) NAFLD activity score in all groups. Data are expressed as mean ± SEM, *n* = 6/group. One-way ANOVA, followed by Tukey post-hoc test. ^a^ *p* < 0.05 vs. Normal group, ^b^ *p* < 0.05 vs. HFD group.

Liver tissues from all the experimental groups were examined histologically to evaluate the NAS score (Figure 7). In this study, the liver tissues from the N group revealed a normal architecture of hepatocytes, well-spaced portal triads and sinusoids. No appearances of fat deposition (micro- or macro-steatosis) were observed in the N group (Figure 7A), while the HFD group showed degenerative changes in hepatocytes along with the presence of high steatosis (micro-and macro-vesicular steatosis), accompanied by inflammatory cells infiltrated particularly around the portal areas (Figure 7B). Administration of orlistat in the HFD + O and OB + O groups showed less hepatic steatosis and inflammation but did not completely reverse them (Figure 7C,D). The NAS in the liver was analyzed and graded (Figure 7E). No signs of MAFLD were found in livers of the N group. By contrast, all animals fed with HFD had a positive NAS. However, the average NAS for HFD + O and OB + O groups reached a maximum of 4 and therefore suggested the presence of simple steatosis. The average NAS in the HFD group was 7, clearly indicating the presence of active NASH (Figure 7E).

## 4. Discussion

In the present study, we demonstrated that the administration of orlistat exerted beneficial effects against metabolic syndrome and oxidative stress in HFD-induced MAFLD rat. Orlistat demonstrated its efficiency in both protective and therapeutic models against MAFLD-related undesirable effects such as insulin resistance, hyperlipidemia, and liver damage. Furthermore, the intake of orlistat also showed a reduction in oxidative stress levels and increases in antioxidant enzymes activities, which might be ascribed to mechanisms involving reduction in the body weight gain and obesity. We also observed the effects of orlistat on the activation of Nrf2/Keap1 signaling pathway in MAFLD rats. Nrf2/Keap1 signaling pathway is a key regulator of oxidative stress and repressor of lipogenesis, which plays a vital role in MAFLD-related metabolic syndrome and oxidative stress.

Body weight gain is primarily determined by food and calorie intakes, and it is an important parameter in evaluating the development of obesity as well as monitoring the effectiveness of certain anti-obesity treatment [5]. In this study, significant increases in the Lee obesity index, body weight gain, and adipose tissues were demonstrated in the HFD group in comparison with the N group, indicating the establishment of obesity in this group. The intake of orlistat in both protective and therapeutic groups significantly reduced the above parameters caused by HFD intake. These benefits could be ascribed to the action of orlistat in the intestinal lumen, which inhibits fat absorption from the intestine [55], thereby reducing the fat mass [29,56,57,58,59]. The inhibition of fat absorption by orlistat is also supported by decreases in serum LDL cholesterol, TC, and TG levels as presented in this study. In addition, a significantly higher calorie intake, which was observed in all the HFD-fed groups compared to the N group signifies a higher fat composition of the HFD regimen used in this study, which consisted of 31% fat compared to only 12% fat in the normal diet [40].

Appetite plays an important role in weight loss, which is usually controlled by many regulators such as the appetite centers in the brain stem and hypothalamus, as well as hormonal signals of the energy status secreted by the gut and periphery [60]. In this study, we observed no significant changes in the food intake among all groups. This might suggest that HFD and orlistat do not affect the appetite or eating behavior and the calorie intake is independent of the amount of food consumed by the animals as previously reported [61]. Our results are in accordance with previous reported studies, which demonstrated no changes in the food intake after 12-week administration of orlistat in obese male rats [34,62]. However, other related studies reported increased appetite after orlistat consumption as shown by increased level of serum ghrelin (gut-derived hormone) in obese women (who received 120 mg orlistat, 3 times/day for 12 weeks) [63], reduced levels of plasma cholecystokinin (CCK) (for inducing fullness and reducing hunger), peptide YY (PYY) and glucagon-like peptide-1-(7–36)-amide (GLP-1) in healthy individuals (received single dose of 120 mg orlistat) [64]. These inconsistencies might be due to the differences in types of subject and doses of orlistat used, as well as the durations of the studies.

Insulin resistance is one of the variables in metabolic syndrome associated with MAFLD and it is defined as a decrease or an insufficient insulin sensitivity in the target tissues, such as in muscle, adipose tissue, and liver towards glucose uptake from the blood [65]. Previous data showed that HFD-fed animals demonstrated reduction in insulin sensitivity [64,65,66,67,68]. This is attributed to the excessive free fatty acids derived from HFD, which inhibit insulin binding, degradation, and function, and hence causes a decrease in glucose uptake from the blood [69,70]. Consistent with the previous reported studies, our finding also showed that rats in the HFD group demonstrated insulin resistance as indicated by significant elevated values of HOMA-IR relative to N group. This is supported with our OGTT findings in HFD group whereby significant increases of glucose concentrations were observed following glucose loading, indicating an impaired insulin function, which could be linked to insulin resistance after prolonged HFD feeding.

Furthermore, previous studies have presented various concrete data on the role of adipose tissue as a key endocrine organ that mediates the metabolic activities of the brain, muscle, and cardiovascular system [71,72]. The adipocytokines such as tumor necrosis factor-alpha (TNF-α), leptin, adiponectin, resistin, and plasminogen activator 1 (PAI-1) released by the adipocytes control appetite, insulin sensitivity, and inflammation, which also take part in the pathogenesis of MAFLD and its progression to NASH [73,74,75,76]. In the present study, we showed that HFD feeding in rats significantly increased the weight of total adipose tissue compared to rats in the N group. In contrast, orlistat administration significantly reduced this parameter as well as increased the insulin sensitivity, which are in accordance with previous studies reported in animal and clinical trials [77,78,79].

Insulin resistance has also been correlated with oxidative stress as demonstrated by Evan et al. [80]. Increased oxidative stress level initiates the activation of serine-threonine kinases activities such as c-Jun N-terminal kinase, which results in phosphorylation of serine residues in the insulin receptor substrate (IRS) protein, which in turn inhibits the effect of insulin signaling pathway [81]. This is in line with our findings in which significant increases in the oxidative stress markers such as TBARS, NO, and PCO levels were shown in the HFD group compared to the N group, suggesting the presence of oxidative stress that may play an essential role to the insulin resistance in HFD group. Nevertheless, orlistat in both protective and therapeutic groups significantly decreased the levels of these oxidative stress markers and insulin resistance.

Lipogenesis comprises conversion of glucose into fatty acids, which in turn is esterified with glycerol 3-phosphate to form TG. The lipogenesis is an insulin- and glucose-dependent pathway activity that is majorly controlled by specific transcription factors, sterol regulatory element binding protein-1c (SREBP-1c), activated by insulin and carbohydrate response element binding protein (ChREBP), which is activated by glucose [82]. Previous study suggests that insulin resistance status is highly related to the alteration of lipid mechanism, accompanied by reduced serum HDL as well as increased LDL and TG levels [83]. In the present study, significantly increased serum TC, TG, and LDL levels and significantly reduced serum HDL level were shown in HFD group. Interestingly, orlistat administration in both protective and therapeutic groups significantly reversed all these undesirable changes. It has been suggested that the anti-hyperlipidemic property of orlistat might also be attributed to its inhibition on TG digestion in the small intestine, thereby reducing the dietary TG hydrolysis, which affects the transfer rate of dietary fatty acids from the small intestine to the lymph before being metabolized in the liver and subsequently transported in the blood [84]. The ability of orlistat in repressing the pancreatic lipase activity in the gastrointestinal tract thus blocks the uptake of lipolysis products, including free fatty acids and monoglycerides, and hence reduces the level of lipids in the blood [85]. In addition, we found a significant increased HDL level in orlistat-treated groups compared to HFD group, which in agreement with previous findings [62,86]. Also, it has been reported that orlistat is able to induce maturation of HDL particles, increase HDL-associated enzymes paraoxonase-1 (PON1) and lipoprotein associated phospholipase A2 (HDL-LpPLA2), resulting in high level of HDL particles [87]. These enzymes linked to HDL are responsible for the antioxidant property exerted by HDL, which might also explain for the decreased levels of oxidative stress markers TBARS, NO, and PCO in the present study.

The liver plays a vital role in regulating the lipid uptake, synthesis, and distribution [88]. An imbalance of these mechanisms can cause excessive accumulation of liver TC and TG, which is accompanied with the presence of steatosis or steatohepatitis (steatosis, hepatocyte hypertrophy, and inflammation) [89]. These prominent features as well as increases in liver weight and adipose tissue are used as prominent features for MAFLD [90,91], and are recognized as the major pathological results of metabolic syndrome [92]. The adipose tissues secrete high levels of adipokines and free fatty acids into the portal vein, which subsequently result in the increased concentration of free fatty acids in the liver tissue [93]. Our present study demonstrated that rats subjected to HFD had significantly increased liver and adipose tissue weights, liver TC, and TG levels, as well as development of micro- and macro-vesicular steatosis, hepatocytes hypertrophy, inflammation, and cell necrosis as evidenced histologically, hence indicated the presence of NASH in this group. Administration of orlistat in both protective and therapeutic groups reversed all these changes significantly. This seems to corroborate with the previous published reports [31,94], suggesting that orlistat may reduce liver lipid accumulation, and hence may alleviate MAFLD and metabolic syndrome.

Liver enzymes are usually used as a sign of liver impairment [5,95] and as surrogate diagnostic markers for MAFLD apart from liver biopsy as a gold standard for MAFLD diagnosis [96]. Any damage in the hepatocytes increases these enzymes activities in the liver before being transported into the bloodstream, and thus increases the enzymes levels in the serum [97]. This coincides with our findings in which increased activities of serum ALT, AST, and ALP enzymes were discovered in HFD rats, indicating liver damage. On the other hand, orlistat administration improved liver function since it normalized the serum activities of these liver enzymes in both protective and therapeutic groups, indicating reduced liver damage in the HFD-induced MAFLD [94,98].

Nrf2 plays a vital role in the maintenance of redox homeostasis and demonstrates its importance in regulating metabolic syndrome and MAFLD [99]. Our results showed increased expression of Nrf2 in the nucleus of the HFD group compared to the N group, which indicates higher translocation of Nrf2 from the cytoplasm to nucleus. However, this study also observed a reduction in antioxidant enzymes (SOD, CAT, GPx, GST, GST, and GR) activities in the HFD group, compared to the N group. Although there was an increase of Nrf2 in the nucleus of HFD group, reduced antioxidant enzymes activities might be a result of increased inactivation or decreased synthesis of the enzymes by the excessive production of ROS [100,101]. Apart from that, orlistat managed to activate the translocation of Nrf2 from the cytoplasm into the nucleus as presented by the higher accumulation of nuclear Nrf2 compared to cytoplasmic Nrf2 following orlistat administration. These results might also explain the significant increased antioxidant enzymes activities observed in the orlistat-treated groups, in comparison with the HFD group by possibly increasing the synthesis of these enzymes. This can be evaluated by assessing their mRNA levels in future study. Furthermore, this study found significantly reduced Keap1 expression in the cytoplasm after orlistat administration, which indicates the reduced inhibitory effects of Keap1 on Nrf2 in these groups, and hence facilitated the translocation of Nrf2 into the nucleus. Previous findings showed that activation of Nrf2 by certain compounds was via inhibitions of Keap1 protein and Nrf2/Keap1 complex [102,103]. The compounds might dominate the Nrf2 binding site in the protein, inhibit the Nrf2/Keap1 complex and subsequently remove Nrf2 from Keap1 to enable Nrf2 nuclear translocation. Nevertheless, the present study did not measure the action of orlistat on the interaction between Nrf2 and Keap1. Hence, it is not known clearly how orlistat promotes the dissociation of Nrf2 from Keap1 which requires further study to elucidate the potential mechanism of action. In addition, apart from immunohistochemistry method, it is also suggested to further confirm the levels of cytoplasmic Nrf2 and Keap1, and nuclear Nrf2 using Western blot technique.

## 5. Conclusions

In summary, the present study demonstrated the protective and therapeutic roles of orlistat against various deleterious effects in MAFLD, including insulin resistance, hyperlipidemia, oxidative stress, and liver injury. These beneficial effects of orlistat may be linked to its ability to reduce body fat deposition and promote the activation of Nrf2, and hence increased the transcriptional expression of Nrf2-targeted antioxidant enzyme genes. This study also highlighted that orlistat did not show significant superior anti-obesity effect when taken either concomitantly or after the induction of obesity. However, a further longer duration study is warranted to elucidate the differences of orlistat administration between these two different models.

## Figures and Tables

**Table 1:** Composition of rat diets.

Nutrient Composition (g/100 g)	Energy (Kcal)	Fat	Carbohydrate	Protein	Ash
Normal diet	318.8	12	64	24	6.9
HFD	516.5	31	46	12	3.8

**Table 2:** Lee obesity index, body weight gain, food intake and calorie intake of rats in the experimental groups.

	N	HFD	HFD + O	OB + O
Lee obesity index	304.60 ± 1.43	334.20 ± 2.66 ^a^	310.00 ± 3.77 ^b^	314.50 ± 2.91 ^b^
Initial body weight (g)	218.20 ± 6.26	233.50 ± 7.53	226.60 ± 6.23	216.80 ± 13.82
Final body weight (g)	352.60 ± 13.68	474.00 ± 12.12 ^a^	403.5 ± 14.44 ^b^	390.80 ± 15.03 ^b^
Body weight gain (g)	93.17 ± 8.97	213.20 ± 12.95 ^a^	159.6 ± 6.92 ^a,b^	152.8 ± 8.31 ^a,b^
Food intake (g/day)	20.79 ± 0.56	21.22 ± 0.82	18.58 ±0.71	19.28 ± 0.66
Calorie intake (kJ/day)	276.50 ± 6.81	458.70 ± 17.72 ^a^	401.40 ± 15.39 ^a^	409.30 ± 18.64 ^a^

Data are presented as mean ± SEM, *n* = 6/group. N, normal control; HFD, high-fat diet; HFD + O, high-fat diet+orlistat 10 mg/kg/day (protective model); OB + O, obese+orlistat 10 mg/kg/day (therapeutic model). One-way ANOVA, followed by Tukey post-hoc test. ^a^ *p* < 0.05 vs. N group, ^b^ *p* < 0.05 vs. HFD group.

**Table 3:** Weights of liver and adipose tissues of rats in the experimental groups.

	N	HFD	HFD + O	OB + O
Absolute liver weight (g)	8.73 ± 0.29	15.74 ± 0.56 ^a^	13.20 ± 0.61 ^a,b^	12.29 ± 0.41 ^a,b^
Relative liver weight (g/body weight)	2.44 ± 0.03	3.44 ± 0.09 ^a^	3.12 ± 0.11 ^a,b^	2.80 ±0.07 ^a,b^
Epididymal adipose tissue weight (g)	2.89 ± 0.14	12.20 ± 1.22 ^a^	5.52 ± 0.82 ^b^	5.20 ± 0.42 ^b^
Peritoneal adipose tissue weight (g)	2.66 ± 0.36	14.14 ± 2.45 ^a^	7.84 ± 1.67 ^b^	5.51 ± 0.32 ^b^
Perirenal adipose weight tissue (g)	0.34 ± 0.03	0.54 ± 0.03 ^a^	0.40 ± 0.02 ^b^	0.41 ± 0.02 ^b^
Total adipose tissue weight (g)	6.87 ± 0.47	26.78 ± 4.34 ^a^	13.11 ± 2.75 ^b^	10.08 ± 1.28 ^b^

Data are presented as mean ± SEM, *n* = 6/group. N, normal control; HFD, high-fat diet; HFD + O, high-fat diet + orlistat 10 mg/kg/day (protective model); OB + O, obese + orlistat 10 mg/kg/day (therapeutic model). One-way ANOVA, followed by Tukey post-hoc test. ^a^ *p* < 0.05 vs. N group, ^b^ *p* < 0.05 vs. HFD group.

**Table 4:** Liver oxidative stress and antioxidants markers.

Parameters	N	HFD	HFD + O	OB + O
TBARS (nmol/mg protein)	2.47 ± 0.07	6.25 ± 0.61 ^a^	2.07 ± 0.27 ^b^	1.82 ± 0.11 ^b^
NO (µmol/g protein)	0.84 ± 0.04	1.20 ± 0.07 ^a^	0.92 ± 0.03 ^b^	0.91 ± 0.06 ^b^
PCO (mmol/mg protein)	0.80 ± 0.05	1.80 ± 0.07 ^a^	0.86 ±0.06 ^b^	0.95 ± 0.11 ^b^
SOD (unit/mg protein)	5.65 ± 0.27	2.12 ± 0.51 ^a^	5.08 ± 0.37 ^b^	4.09 ± 0.50 ^b^
CAT (unit/mg protein)	28.28 ± 3.56	6.70 ± 1.14 ^a^	16.45 ± 0.80 ^b^	21.77 ± 3.03 ^b^
GPx (unit/mg protein)	2.45 ± 0.20	0.79 ± 0.16 ^a^	2.416 ± 0.18 ^b^	1.79 ± 0.11 ^a,b^
GST (unit/mg protein)	5.49 ± 0.64	1.47 ± 0.09 ^a^	4.26 ± 0.34 ^b^	4.62 ± 0.62 ^b^
GSH (nmol/mg protein)	3.29 ± 0.08	2.37 ± 0.10 ^a^	3.06 ± 0.06 ^b^	2.91 ± 0.19 ^b^
GR (unit/mg protein)	15.36 ± 0.46	11.40 ± 0.36 ^a^	15.55 ± 0.60 ^b^	14.61 ± 0.87 ^b^
TAC (nmol/mg protein)	83.11 ± 2.59	65.92 ± 0.56 ^a^	84.11 ± 3.04 ^b^	83.70 ± 6.51 ^b^

Data are expressed as mean ± SEM, *n* = 6/group. N, normal control; HFD, high-fat diet; HFD + O, high-fat diet + orlistat 10 mg/kg/day (protective model); OB + O, obese + orlistat 10 mg/kg/day (therapeutic model); TBARS, thiobarbituric acid reactive substances; NO, nitric oxide; PCO, protein carbonyl; SOD, superoxide dismutase; CAT, catalase; GPx, glutathione peroxidase; GST, glutathione S-transferase; GSH, glutathione; GR, glutathione reductase; TAC, total antioxidant capacity. One-way ANOVA, followed by Tukey post-hoc test. ^a^ *p* < 0.05 vs. N group, ^b^ *p* < 0.05 vs. HFD group.

## Data Availability

The data are presented within the paper. Additional raw data are available on request from the corresponding author.
